# Single-Dose *IncobotulinumtoxinA* in the Treatment of Early-Stage Knee Osteoarthritis: Results from a Preliminary Single-Arm Clinical Trial

**DOI:** 10.3390/toxins17050216

**Published:** 2025-04-25

**Authors:** Sofia Durán-Hernández, Norma E. Herrera-González, Nayar Durán-Hernández, Martha Carnalla, Manuel de Jesús Castillejos-López, Citlaltepetl Salinas-Lara

**Affiliations:** 1 Hospital General Tacuba, Instituto de Seguridad y Servicios Sociales de los Trabajadores del Estado (ISSSTE), Profesor de Asignatura, Facultad de Medicina, Universidad Nacional Autónoma de México (UNAM), Mexico City 04360, Mexico; 2 Escuela Superior de Medicina, Instituto Politécnico Nacional (IPN), Mexico City 11340, Mexico; neherrera@gmail.com; 3 Pitzer Laboratory of Osteoarthritis Research, German Rheumatism Research Center (DRFZ), Leibniz Institute, 10117 Berlin, Germany; 4 Centro de Investigación en Salud Poblacional, Instituto Nacional de Salud Pública, Cuernavaca 62100, Mexico; martha.carnalla@insp.mx; 5 Investigador, Unidad de Vigilancia Epidemiológica, INER Ismael Cosío Villegas, Mexico City 14080, Mexico; 6 Departamento de Neuropatología, Instituto Nacional de Neurología y Neurocirugía (INNN), Mexico City 14269, Mexico; citlalsalinas69@gmail.com; 7 Red MEDICI Carrera de Médico Cirujano, FES Iztacala, Universidad Nacional Autónoma de México (UNAM), Mexico City 54090, Mexico

**Keywords:** osteoarthritis, knee OA, botulinum toxins, pain, case series, *IncobotulinumtoxinA*

## Abstract

Osteoarthritis (OA) is the most prevalent rheumatologic disease and a leading cause of years lived with disability worldwide. There are no disease-modifying drugs available to treat it. This study aimed to evaluate the effect of a single dose of 100U botulinum neurotoxin-A (BoNT-A) in patients with early knee OA. We designed a single-arm preliminary clinical trial in patients diagnosed with knee OA (KOA) grades I and II. 45 Patients received a single dose of 100U *IncobotulinumtoxinA* in the retro-patellar bursa and received nutritional and physical rehabilitation indications. Patients were evaluated at baseline and at days 5, 30, 60, and 90 after injection. The primary outcome was the reduction in pain using the visual analog scale (VAS). Knee function was evaluated using the Western Ontario and McMaster Universities Osteoarthritis Index (WOMAC). We assessed secondary adverse effects and measured muscular strength in every consultation. Descriptive endpoint summaries and a generalized linear random-effect model were used to evaluate changes in each follow-up time compared to baseline. *IncobotulinumtoxinA* treatment significantly (*p* < 0.001) reduced pain in all treated patients at day 90 compared to day 0. Patients showed a significant reduction in total WOMAC score (*p* < 0.001), from a mean baseline of 44.6 (95% CI; 41.4, 47.8) to 4.4 at day 90 (95% CI; 0.2, 0.3). Our results show that *IncobotulinumtoxinA* applied in the retro-patellar bursa is a safe and effective treatment for pain in patients with early-stage KOA, offering a potential alternative for symptomatic control in KOA.

## 1. Introduction

Osteoarthritis (OA) is the most common arthropathy worldwide and the 11th-highest contributor to global disability in adults [1,2]. Knee OA (KOA) is the most common presentation of the disease, with a 3.8% age-standardized prevalence worldwide [3]. Global OA prevalence and its annual incidence rate increased by 9.3% and 8.2%, respectively, between 1990 and 2017, a rise attributed to the aging of the global population [3]. OA is a multifactorial and heterogeneous disease whose pathogenesis is not entirely understood. OA affects the entire joint, leading to degradation of the articular cartilage, sclerosis of the subchondral bone, inflammation of the synovial membrane, formation of osteophytes, and degeneration of menisci, ligaments, and the joint capsule [4]. Obesity, aging, and joint trauma are among the main risk factors leading to joint damage and the initiation of a local immune response that develops into low-grade chronic inflammation, ultimately causing OA [5].

Pain is the main OA symptom, and it is also the most disabling. Therapy decision-making depends on the patient’s pain and secondary functional limitations [6]. This symptom is clinically evaluated using patient questionnaires like the visual analog scale (VAS) to assess perceived pain in the joint of interest in patients using a numerical continuum [7]. To study other characteristics of knee osteoarthritis, the Western Ontario and McMaster Universities Osteoarthritis (WOMAC) Index evaluates articular function, perceived rigidity, and pain by asking 24 questions using a Likert scale format [8,9,10].

Pain itself compromises the recovery of the patients by limiting their movement and function and their ability to follow rehabilitation indications [10]. Identifying the mechanism leading to increased pain in OA is a subject of ongoing research [11]. To date, OA treatments are mainly symptomatic, using anti-inflammatory drugs and painkillers [6].

Cartilage loss is the histopathological landmark of the disease and has been considered a potential culprit of pain. This association led to therapy goals that aimed at both protecting the cartilage and, as a consequence, decreasing the pain perceived by the patient [11]. However, this correlation might be erroneous. A recent study using patient information from the Osteoarthritis Initiative (OAI) used magnetic resonance imaging (MRI) to evaluate cartilage loss and studied its correlation to perceived pain over a 36-month period [11]. Surprisingly, it was found that pain is only partially correlated to cartilage loss. However, the analysis of the characteristics of the synovial membrane showed that worsening of synovitis better explained pain over the follow-up period [11]. These data contradict the common idea that cartilage loss is the primary cause of pain increase in OA and suggest that chondroprotection and analgesia in OA therapy are not equivalent endpoints, requiring distinct therapeutic strategies [11].

Unfortunately, MRI evaluation is not generally accessible, and the evaluation of the joint cartilage, synovium, ligaments and menisci is not normally performed in clinical practice. Instead, the Kellgren and Lawrence radiographic scale is used. This evaluation tool uses antero-posterior knee radiographies and provides information on joint alignment and the proximity of the tibial and femoral articular surfaces, limiting the diagnosis of early grades of KOA or affected joint tissues other than bone [11,12,13]. A recent meta-analysis found that patients with KOA have greater local pain sensitivity than healthy counterparts and that pain sensitization by quantitative sensory testing is highly correlated to symptom severity but not to radiographic severity [14], suggesting that a mechanism leading to increased pain can be present in low-grade KOA patients.

The development of therapies against pain should be prioritized and separated from chondroprotective goals for approval by medical regulatory organizations. According to the new Osteoarthritis Research Society International (OARSI) guidelines, initial non-pharmacologic treatment for KOA should include arthritis education, weight management, land-based exercise, and walking aids when needed [15]. Intra-articular (i.a.) steroidal therapy is recommended only during acute pain due to the risk of cartilage loss observed with prolonged treatment [15], while i.a. hyaluronic acid (HA) treatment is recommended for patients with persistent symptoms for long-term analgesia [15]. The therapeutic options are limited to symptomatic pain control since no disease-modifying therapy has been developed [10]. There is still a need for better long-term analgesics, particularly in patients who are not candidates for surgical treatment.

One potential candidate for knee pain control is botulinum toxin type A (BoNT-A) [16]. Analgesia was first observed as a side effect concomitant to muscle relaxation after BoNT application [17,18]. BoNT-A provides analgesic effects in pain disorders such as chronic migraine [19], trigeminal neuralgia [20], peripheral neuropathic pain [21,22,23], and other pain syndromes [24,25]. The analgesic effect produced by BoNT appears to be multifactorial, by blocking the release of pain-related neurotransmitters [16,26], by blocking the release of excitatory neurotransmitters in the dorsal root ganglions after being retrogradely transported in peripheral administration [27], and potentially, by indirect desensitization of peripheral nociceptors [24].

Clinical use of i.a. BoNT-A for refractory joint pain was first documented in a case report series of patients suffering from OA, rheumatoid and psoriatic arthritis (PsA) [28], showing no adverse effects as well as long-term analgesia and functional improvement of the affected joints. The BoNT effect was then tested in murine arthritis models, finding analgesia and increased physical activity after the i.a. administration of both BoNT-A [29] and BoNT type B [30] in chronic arthritis models, but not in acute models. Furthermore, animal studies provided evidence of analgesic effects in doses that did not impair limb movement or muscular function [29].

In a subsequent single-center clinical trial using i.a. injections of BoNT-A, improved quality of life and superior analgesia were reported when compared to i.a. saline combined with lidocaine in patients with refractory pain due to shoulder arthritis [31]. In KOA, studies using i.a. BoNT-A have shown a similar safety profile and better analgesia than i.a. corticosteroids [32], better pain relief and functional improvement than patients receiving only education for arthritis care [33], and regimes using i.a. HA or i.a. saline injections plus therapeutic exercise [34,35].

Despite this promising first evidence, conflicting results have been recently published. A post hoc analysis restricted to patients with KOA and nociceptive pain showed a significant improvement in all WOMAC index criteria and reduced intake of rescue analgesic medication after i.a. BoNT-A treatment [36]. This indicates that patients with nociceptive pain are a population that could benefit from this treatment [36]. However, a subsequent study in KOA patients with nociceptive pain showed no significant difference in pain relief eight weeks after the application of i.a. BoNT compared to i.a. saline solution; that is, both displayed similar analgesic effects [37]. Finally, similar results were reported in patients with KOA with Kellgren and Lawrence grades I and II, showing no significant difference in pain or function 12 weeks after treatment between patients treated with i.a. triamcinolone, i.a. BoNT-A (100U), or i.a. saline solution, suggesting similar effectiveness of the three treatments at this time point [38]. However, this study found an improved effect of i.a. triamcinolone in pain relief, knee function, and synovitis compared to saline and BoNT-A at the 4-week time point. In all reviewed studies, i.a. BoNT-A showed similar safety and analgesia to conventional therapy and placebo (saline) injections [31,32,33,34,36,37,38,39,40,41,42].

There is a great need for new therapeutics to control pain in KOA, particularly in patients who are not candidates for surgical treatment and need long-term pain control [10]. Total knee arthroplasty (TKA) is usually contraindicated in young patients who live with obesity and metabolic comorbidities [10], a constellation particularly common in the Hispanic population of our clinical settings. TKA is not indicated or convenient in patients with early-grade KOA, leaving this population with prolonged functional limitation until surgery is possible while complicating their physical rehabilitation due to pain [10]. Here, we developed the current study considering these gaps in the literature by including patients with early KOA to test the safety of i.a. BoNT-A injection (*IncobotulinumtoxinA*, Xeomeen, Merz Therapeutics GmbH, Frankfurt am Main, Germany) in a Hispanic population and evaluated its effect on pain reduction, muscular strength, and articular function.

## 2. Results

We enrolled a total of 45 patients; all completed the 90-day follow-up period. Table 1 summarizes their sociodemographic and clinical characteristics. Most patients were women (37, 82.2%) with an average age of 59. All patients had early OA, with Kellgren and Lawrence grades I (22.2%) and II (77.8%). Most of the patients were obese (35.6%) or overweight (60%) (Table 1). Regarding affected joints, 43 patients had bilateral KOA; one had unilateral left KOA, and one had right KOA. Twenty-seven percent of the patients (n = 12) achieved nutritional and rehabilitation compliance at the end of the follow-up period. During the follow-up period, no patient reported or showed signs of adverse effects derived from the application of the treatment.

### 2.1. Therapeutic Effect in Pain, Stiffness, and Function (WOMAC)

The summary of WOMAC results for all patients for days 0, 30, 60, and 90 after treatment is shown in Table 2. Of note, due to the application characteristics of the WOMAC questionnaire, these results refer to the overall perceived effect found by the patient in the treated knees, not separating the results by a specific limb. We found a significant 56.7% reduction (*p* = 0.003, −25.24 points) in the total WOMAC score at day 30 after therapy compared to baseline. This was further reduced at day 90 (−87.4% or −38.97 points compared to baseline). All WOMAC components showed a similar reduction over the follow-up period (Table 2).

### 2.2. Therapeutic Effect in Referred Pain (VAS)

Mean visual analog scale (VAS) score was significantly (*p* < 0.0001) reduced 5 days after treatment compared to baseline: −2.43 points, 39.7% decrease in right knees, and −2.2 points, 35.5% decrease in left knees. Pain kept decreasing until the day 90 follow-up: 4.96 points or 81.2% decrease in the right knee (*p* < 0.0001) and −5.09 points, 81.5% decrease (*p* < 0.0001) in the treated left knees (Table 2).

### 2.3. BoNT-A Effect on Muscular Strength of the Lower Limb

The mean basal extension strength of the right knee was 30.6 kg (SD 11.6), while the left knee was 28.9 kg (SD 11.9). The mean basal flexion strength was 18.7 kg (SD 7.6) for the right knee and 18.5 kg (SD 7.3) for the left knee. Patients showed an average strength increase of 49.2% (15.1 kg) in extension of the right knee and 43.0% (12.5 kg) in the left knee at day 90 compared to baseline. Flexion strength in the same period showed a significant increase of 60.5% (11.3 kg) in the right knee and 42.2% (7.8 kg) in the left knee (Table 2).

### 2.4. Generalized Linear Random Effect Model

**Figure 1:**
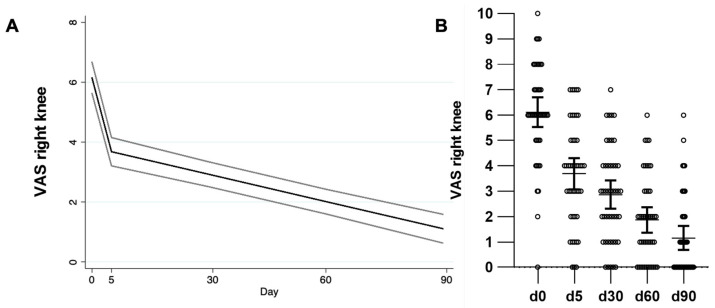
Visual analog scale (VAS) of the right knee. (**A**) GLM showing VAS reduction and 95% CI of the right knee over time are shown, adjusted by weight and compliance. (**B**) Datapoints, mean, and 95% CI are shown. GLM: generalized linear model. CI: Confidence intervals.

**Figure 2:**
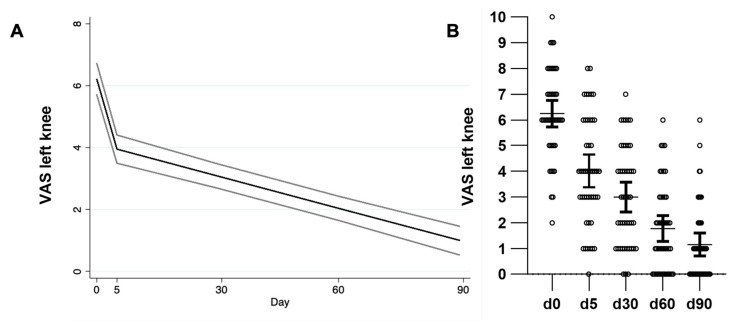
Visual analog scale of the left knee. (**A**) GLM showing VAS and 95% CI of the left knee over time are shown, adjusted by weight and compliance. (**B**) Datapoints, mean, and 95% CI are shown. GLM: generalized linear model. CI: Confidence intervals.

**Figure 3:**
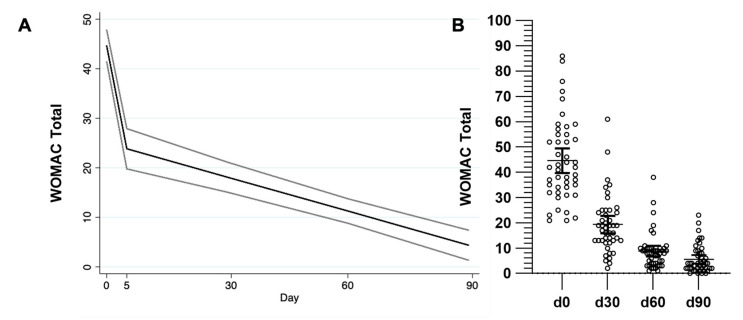
Total WOMAC index. (**A**) GLM showing WOMAC index reduction and 95% confidence intervals over time, adjusted by weight and compliance. (**B**) Datapoints, mean, and 95% CI are shown. GLM: generalized linear model. CI: Confidence intervals.

Table 3 shows the coefficients of the variables included in the model for each outcome adjusted for age, weight, and compliance. For the right knee, by day 5 the VAS score showed a significant mean reduction of 2.3 points (95% CI −2.8, −1.8), and for each additional day, the mean reduction was 0.03 points (95% CI −0.04, −0.02) (Figure 1, Table 3). For the left knee, by day 5, the VAS score showed a significant mean reduction of 2.1 points (95% CI −2.6, −1.6), and for each additional day, the mean reduction was 0.03 points (95% CI −0.04, −0.03) (Figure 2, Table 3). By day 5, the WOMAC score showed a significant mean reduction of 19.7 points (95% CI −24.2, −15.1) adjusted by age, weight, and compliance. For every day further, the mean reduction was 0.2 points (95% CI −0.3, −0.2) (Figure 3, Table 3).

## 3. Discussion

Patients displayed an important reduction in pain in both knees and a significant improvement in the WOMAC index score after a single intra-articular dose of 100U of *IncobotulinumtoxinA*. This effect was noted only five days after the i.a. injection and continued to decrease throughout the 90-day follow-up. This early analgesic effect cannot be explained by weight loss or physical therapy. The reduction in pain by day five (39.7% right knee, 35.5% left knee) was comparable to previously reported data at week 1, both in a clinical trial with a control group receiving educational indications [33] and approximately double the effect reported in an i.a. placebo-controlled clinical trial [37].

In our study, by the end of the 90-day follow-up, patients showed a sustained reduction in the referred pain (−81.2% in the right knee and −81.5% in the left). This pain reduction is similar to the one found in a clinical case series [41], a preliminary single-center study using *OnabotulinumtoxinA* 100U [33], and a trial combining conventional rehabilitation measures with hyaluronic acid, *OnabotulinumtoxinA* 100U, or i.a. saline solution [34]. Recently, a study with a single-dose *AbobotulinumtoxinA* 250U showed similar pain reduction scores to ours 4 weeks after treatment [40].

In our study, physical therapy was coupled with our therapeutic intervention. A couple of studies have compared the analgesic effect of BoNT in combination with physical therapy regimens. Rezasoltani et al. showed that a single-dose *AbobotulinumtoxinA* (250U) offered a significant additional analgesic effect when compared to physiotherapy alone in both the VAS and knee osteoarthritis outcome score (KOOS) [35]. Notably, this additional analgesia was also observed using 3 monthly doses of dextrose 20% + 2% lidocaine, but no benefit was obtained using monthly doses of HA. Bao et al. evaluated physical therapy in addition to i.a. single-dose injections of saline solution, *OnabotulinumtoxinA* (100U), or HA, showing that only the BoNT-A injection offered an additional benefit in both VAS and WOMAC score reduction at 4 and 8 weeks after intervention [34]. In both studies, authors hypothesize that analgesia obtained by i.a. BoNT injections synergizes with physical exercise therapies, improving knee functionality and reducing pain. This synergic effect was assessed by a recent scoping review including eleven studies that used i.a. injection therapies (steroid, HA, or BoNT) in combination with physical therapy and concluded that there is still a need for novel clinical trials using BoNT and HA interventions [43]. In our study we could not compare the effects to physical therapy alone; however, we obtained similar analgesic effects as previous studies evaluating BoNT-A in combination with therapeutic exercise regimes [34,35].

There is conflicting evidence published on using i.a. BoNT-A injections to treat KOA. Three placebo-controlled studies showed no additional pain reduction when comparing i.a. injections of BoNT and saline solution [36,37,38], being both the placebo and BoNT treatments analgesic for the patients. These studies did not include baseline physiotherapy as a complement to i.a. injections and could have omitted the potential analgesic benefit of i.a. injection therapies in the context of treatment as suggested by clinical guidelines for KOA treatment, which include education and exercise [15,44]. This should be properly assessed in future studies to test the analgesic effectiveness of i.a. injections with or without physical therapy.

The surprising analgesic effects obtained when including an i.a. placebo group have not only been observed in trials assessing novel therapies. Recently, two distinct meta-analyses showed that i.a. saline solution injection has a similar analgesic effect as i.a. HA and corticosteroid treatments in KOA [45], and similar analgesic and functional effects to commonly used therapies in hip OA [39]. The potential analgesic effect of i.a. saline solution is an ongoing research topic with important implications in placebo-controlled clinical trials. These data indicate the necessity of including an i.a. placebo group in studies assessing injection therapies after testing the safety of a new drug. However, testing safety should be performed in preliminary pilot studies, particularly in drug repurposing trials, such as the one we conducted for the first time for KOA using *IncobotulinumtoxinA*.

Patients in our study reported lower knee functional impairment measured by WOMAC score after treatment with *IncobotulinumtoxinA* in the observed time points 30, 60, and 90 days after injection, compatible with previously reported data [32,33,34,40]. This functionality improvement aligns with the greater muscular strength found in our cohort. Patients displayed a sustained increase in flexion and extension strength in both limbs, showing a positive effect of the rehabilitation program on the muscular function of the patients and the absence of muscular-weakening effects after i.a. BoNT-A injection. This goes in line with previous trials using other commercially available botulinum toxins (*OnabotulinumtoxinA* and *AbobotulinumtoxinA*), where toxins showed a comparable safety profile to i.a. steroid injection [32], education-only treatment [33], i.a. HA [34], and i.a. saline injections [34,36,37]. However, our study adds important safety data using *IncobotulinumtoxinA* for the first time in i.a. knee applications as well as the inclusion of a Hispanic population cohort for BoNT-A toxin trials.

The analgesia observed in our study after applying i.a. *IncobotulinumtoxinA* could be explained by the inhibition of neurotransmitter release at peripheral neuron synapses in the synovium [37]. BoNT-A produces this effect by impeding the union of transport vesicles to the cellular membrane of peripheral neurons, mitigating pain sensitization [16,21]. Additionally, a central effect of the drug has been suggested by the retrograde transport that the toxin displays in the dorsal root ganglion with further transcytosis to afferent neurons in the brain [16]. Both peripheral and central effects suggest that BoNT could be used in chronic pain treatment.

### Limitations of This Study

This study has some limitations. First, we did not include a placebo injection group due to the preliminary nature of this cohort. Hence, we cannot exclude that the analgesia observed is attributable to the intra-articular injection alone. Second, individuals with overweight and obesity were predominant in our population of study, which might not represent the situation of KOA patients elsewhere. Third, the sole effect of weight loss and physical activity was not evaluated separately. However, we included compliance to medical indications in our generalized linear model to discern the impact of those interventions on the outcome. Fourth, observation of the analgesic effect in our study was limited to 90 days; a longer follow-up is needed to observe the duration of the anti-nociceptive effect of the toxin. Our study has the strengths of a one hundred percent follow-up and the evaluation of the intervention performed in a clinical scenario, including baseline rehabilitation therapy.

There is a great need for new therapeutics to control KOA symptoms. Most studies using i.a. BoNT therapy have included individuals with advanced grades of joint damage and refractory pain [31,40,41,42], skewing the population of the study towards patients with difficult pain control and complicating the possibility of proving the effectiveness of a new treatment in the overall KOA population. Further studies are needed to test the potential therapeutic use of BoNT-A in placebo-controlled trials considering baseline rehabilitation treatment.

## 4. Conclusions

In conclusion, a single intra-articular dose of 100 U of *IncobotulinumtoxinA* in patients with KOA decreased perceived pain and improved the WOMAC index without any side effects reported ninety days after its application. BoNT-A may offer an alternative treatment for patients with comorbidities, those who cannot tolerate NSAID intake, or those who do not improve with conventional pharmacological treatment. BoNT-A provides a safe, tolerable, and lasting therapeutic effect. Future clinical studies comparing BoNT-A treatment with intra-articular saline solution injection, conventional therapy with intra-articular steroids, and groups assessing only nutritional and rehabilitation indications are needed to properly discern a potential placebo effect and consider the baseline therapeutic contribution of commonly used therapy in rehabilitation services. This study adds evidence on the safety and effectiveness of BoNT-A in pain reduction in patients with early OA, which may be an alternative treatment for patients who do not respond to conventional pharmacologic treatment.

## 5. Materials and Methods

### 5.1. Study Design and Patients

We conducted a preliminary single-arm clinical trial in a single rehabilitation center assessing the effect of 100U i.a. *Incobotulinumtoxin* A on pain management and joint function of patients with KOA over 12 weeks. 45 patients were enrolled, all diagnosed with KOA grades I and II according to the American College of Rheumatology guidelines and the Kellgren and Lawrence radiologic scale evaluated in knee radiographs taken 1 to 4 weeks prior to initial evaluation [12]. All patients were referred to the rehabilitation service due to the failure to provide analgesia by their primary healthcare provider. This study was approved by the local Bioethics Committee at the Tacuba General Hospital (Registration number: 017-13). All participants were informed about the study before signing the informed consent. Declining to participate in this study did not affect the patient’s right to receive conventional rehabilitation therapy. All patients completed the 90-day follow-up period.

### 5.2. Intervention

A single 100 U dose of *IncobotulinumtoxinA* (Xeomeen, Merz Therapeutics GmbH, Frankfurt am Main, Germany) was diluted in 1 mL of 0.9% sterile saline solution. BoNT-A was applied in the retro-patellar bursa of the affected knee. Briefly, patients were placed in a supine decubitus position with the knee flexed between 10 and 20° using a small cushion under the articulation. We performed a lateral retro-patellar injection using a 1 ½”, 27G needle. Following needle insertion, gentle synovial fluid aspiration was performed to confirm successful placement inside the bursa, and the drug was slowly injected.

All patients were given indications to reduce weight based on the Mexican clinical practice guidelines for the prevention, diagnosis, and treatment of overweight and obesity [46]. Additionally, patients were instructed to do a daily rehabilitation routine of the lower limbs. This routine was based on the Mexican clinical practice guideline for the diagnosis and treatment of knee osteoarthritis [44].

### 5.3. Clinical Evaluation

Patients were evaluated at baseline (day 0), days 5, 30, 60, and 90 after the *IncobotulinumtoxinA* injection. Pain intensity was determined using the visual analog scale (VAS). Knee stiffness, pain, and functionality were assessed using the WOMAC Index. Higher scores in this tool indicate a worse functionality and perception of pain or stiffness in the patients. All questionnaires were self-applied by the patients while supervised by a physician. Weight, height, and lower-limb muscle strength (DS2 110 digital force gauge, IMADA Inc., Northbrook, IL, USA) were measured at days 0, 30, 60, and 90. Adverse effects were questioned and explored in every medical consultation, including knee pain, headache, joint effusion or swelling, muscular weakness, and inflammation of the injection site.

### 5.4. Statistical Analysis

Baseline characteristics and outcomes were described by means, standard deviation, percentages, and frequencies. To evaluate data distribution, we used the D’Agostino Pearson normality test. All values followed a normal distribution. Considering that the same patient was evaluated in multiple occasions after a single treatment intervention, repeated measures one-way ANOVA with Geisser–Greenhouse correction was conducted with a subsequent Dunnett’s multiple comparison test.

We fitted three generalized linear random-effect models with right knee pain, left knee pain, and total function as the outcomes. The three models were adjusted for age, weight (kg), and compliance. An interaction term of toxin and day was included to evaluate the intervention effect as an approach to evaluate the spline observed on day 5. The weight measurement on day 5 was not taken; thus, it was modeled with a generalized linear model for imputation using sex, age, and toxin intervention. We used weight and muscular strength information on day 90 after treatment as a proxy for compliance with nutritional indications and exercise recommendations. Compliance was defined as a 6 kg reduction or a 15 kg muscle strength increase at day 90. Descriptive analyses and outcome comparisons were made in GraphPad Prism version 8.4.3 for macOS (GraphPad Software, Boston, MA, USA). Analyses for the generalized linear model were performed in Stata v14 (College Station, TX, USA: StataCorp LLC). Statistical significance was set at a *p*-value below 0.05.

## Figures and Tables

**Table 1:** Sociodemographic and clinical characteristics of the patients with early-stage knee osteoarthritis treated with a single dose of *IncobotulinutoxinA*.

Variable		n (%)
Sex	Women	37 (82.25)
	Men	8 (17.8%)
Kellgren and Lawrence	Grade I	10 (22.2%)
	Grade II	35 (77.8%)
Age		59.3 (18, 78) *
BMI	Normal	2 (4.4%)
	Overweight	27 (60%)
	Obese	16 (35.6%)
Comorbidities	Diabetes	1 (2.2%)
	Hypertension	10 (22.2%)
	Diabetes and Hypertension	6 (13.3%)
		n = 45

These patients are considered to be early-stage KOA. BMI: body mass index. * Mean age of the cohort and the minimum and maximum ages of the patients are shown.

**Table 2:** Descriptive endpoints at baseline, day 5, 30, 60, and 90 in patients with early-stage knee osteoarthritis treated with a single dose of *IncobotulinutoxinA*.

Days	0	5	30	60	90
VAS right mean, (SD)	6.11 (1.94)	3.68 (2.03) *	2.86 (1.87) **	1.86 (1.67) **	1.15 (1.58) **
VAS left mean, (SD)	6.24 (1.72)	4.02 (2.105) *	3 (1.93) **	1.77 (1.67) **	1.15 (1.49) **
WOMAC					
Total	44.57 (16.3)	-	19.33 (11.33) *	8.82 (7.15) **	5.6 (5.53) **
Pain	17.95 (5.25)	-	8.02 (3.87) **	4.42 (1.97) **	2.84 (1.9) **
Stiffness	3.8 (1.63)	-	1.28 (1.14) **	0.51 (0.69) **	0.31 (0.55) **
Function	31.66 (12.19)	-	14 (8.8) *	6.06 (5.96) **	3.84 (4.6) **
Extension right, (kg, SD)	30.62 (11.53)	-	39.06 (14.52) *	41.93 (12.62) **	45.71 (13.96) **
Flexion right (kg, SD)	18.66 (7.61)	-	29.94 (9.04) **	26.79 (9.24) **	29.95 (11.91) **
Extension left, (kg, SD)	28.91 (11.85)	-	38.84 (15.33) **	40 (11.69) **	41.37 (11.57) **
Flexion left (kg, SD)	18.5 (7.34)	-	24.33 (10.09) **	24.09 (7.91) **	26.31 (11.06) **

Mean and standard deviation (in parentheses) are shown for all results. For VAS, the assessment was asked to each patient separately for each knee. The WOMAC questionnaire summarizes the patient’s symptoms for the most symptomatic knee. The statistical significance values displayed are comparisons between each time point relative to the baseline. Normally distributed variables (WOMAC pain), RM-one-way-ANOVA with Geisser–Greenhouse correction, and Dunnett’s multiple-comparison test were performed. For comparisons of groups that did not follow a normal distribution, the Friedman test with Dunn’s multiple-comparison test was performed. Significance: * *p* < 0.05; ** *p* < 0.0001.

**Table 3:** Generalized linear model. Mean reductions in VAS in the right and left knees and overall WOMAC in patients with early-stage knee osteoarthritis treated with a single dose of *IncobotulinutoxinA*.

		Toxin	Toxin * Day
VAS right knee * n = 45	Coef.	−2.31 **	−0.03 **
	95% CI	−2.81, −1.8	−0.04, −0.02
VAS left knee * n = 44	Coef.	−2.08 **	−0.03 **
	95% CI	−2.60, −1.57	−0.04, −0.03
WOMAC * n = 45	Coef.	19.66 **	−0.23 **
	95% CI	−24.21, −15.11	−0.29, −0.17

Generalized linear model, VAS: visual analog scale. Compliance was defined as a 6 kg reduction or 15 kg muscle strength increase at day 90. * Model adjusted for age, weight, and compliance. ** *p* < 0.0001.

## Data Availability

The original contributions presented in the study are included in the article, further inquiries can be directed to the corresponding authors.
